# Brushing motion caused no microcracks: a micro-computed tomography study

**DOI:** 10.1007/s00784-025-06253-0

**Published:** 2025-03-10

**Authors:** Deniz Yanık, Şelale Özel, Fügen Dağlı Taşman Cömert

**Affiliations:** 1 Faculty of Dentistry, Department of Endodontics, Süleyman Demirel University, Isparta, Türkiye; 2 Faculty of Dentistry, Department of Oral and Maxillofacial Radiology, Altınbas University, İstanbul, Türkiye; 3 Faculty of Dentistry, Department of Endodontics, Near East University, Mersin 10, North Nicosia, Türkiye

**Keywords:** Brushing motion, Endodontics, Dentinal defects, Microcrack, Micro-CT, Reciprocating motion

## Abstract

**Objective:**

We evaluated the effect of brushing motion on microcrack formation in round distal canals after using multi-file rotary(MFR), single-file rotary(SFR), and single-file reciprocation(SFRc) systems via micro-computed tomography(micro-CT).

**Materials and methods:**

Thirty-six mandibular molars were used. Samples were allocated according to files and preparation patterns (*n* = 12); pecking (P) and brushing (B): Group-MFR-P, Group-MFR-B, Group-SFRc-P, Group-SFRc-B, Group-SFR-P, Group-SFR-B. MFR was ProTaper Next, SFR was TruNatomy, and SFRc was WaveOne Gold. Mesial and distal were prepared using pecking motion, and additional brushing motion. Brushing motions were performed after the pecking motions with 6 strokes. Pre-and-post-instrumentation scans were obtained. Wilcoxon, Krukal-Wallis, and Mann-Whitney-U were performed.

**Results:**

No differences were between pre-and-post-instrumentation scans (*p* > 0.05). Post-instrumentation microcracks were not different in Group MFR-P and Group MFR-B, Group SFRc-P and Group SFRc-B, Group SFR-P and Group SFR-B (*p* > 0.05).

**Conclusion:**

The brushing motion followed by the pecking motion did not cause microcracks. None of the file systems examined in the study induced microcracks.

**Supplementary Information:**

The online version contains supplementary material available at 10.1007/s00784-025-06253-0.

## Introduction

In the earlier stages, during the use of file systems, lateral instrumentation was not recommended but passively central use [1]. However, in the ongoing process of using engine-driven instruments, to enhance instrumentation efficiency, brushing motion gained acceptance in endodontic practice [2]. In the literature, this term, expressed as *‘brushing motion’*, *‘brush-like motion’ ‘lateral brushing movement’*, or ‘*brushing working motion’* meant the instrumentation of the lateral walls of the canal with engine-driven file systems. Brushing motion cuts the dentin during the outstroke and is generally performed on the oval canal morphology [3]. With the removal of a similar amount of dentin from all aspects of the canal walls, brushing motion presents an opportunity to maintain the canal morphology, meanwhile, by pursuing thin dentin at the danger zone [4]. The outward brushing motion did not apply extra torque to the instrument when compared to the inward pecking motion and it was concluded that it was a safer technique [5]. On the other hand, when considering the dentin tubule penetration of microorganisms for more proper microbial control, brushing motion is prominent. During the shaping of the canals with engine-driven instruments, *the pecking motion* is performed to reach the apical of the root, it is performed in 3 or more steps with 2–3 amplitude according to the working length [6, 7]. For analysis of the shaping ability or microcrack formation of different instruments, files are applied with pecking motion [8–13].

Microcracks refer to plastic deformation, which is permanent damage, without displacement or separation occurs in the structural integrity. Cracks or fractures may pose problematic clinical scenarios in terms of restorative procedures due to their various elongation or orientation [14]. In earlier years microcrack was associated with vertical root fracture (VRF). Although over the following years, the mismatch between the prevalence of VRF and microcrack frequency weakens this relationship [6, 7], the presence of microcracks remains an important issue for microbial control, and even if they do not proceed to the VRF, they may cause pain, bone loss, or malfunction of the tooth [6].

There are many methods including cross-section analysis and stereomicroscope with or without dye staining [6], endoscopy [15], scanning electron microscopy [16], transmission electron microscopy [17], optical coherence tomography [13], and micro-computed tomography (micro-CT) [10, 18], synchrotron radiation-based micro-CT (SRCT) [19], and transillumination [20] for the analysis of dentinal microcracks. Foremost, micro-CT began a new era with lower limitations of an in vitro design and was highly accepted in endodontic society for detecting microcracks [7].

In literature, numerous studies analyzed microcrack formation related to using various engine-driven endodontic instruments [8–13, 21–25]. However, there is no comprehensive data about the influence of the brushing motion on the formation of dentinal microcracks, although brushing motion is widely applied to canals during instrumentation.

In in vitro studies that used extracted teeth, the composition and structure of dentin are considered as an important laboratory parameter that should be standardized. The amount of collagen and chemical composition affect dentin elasticity and brittleness [26], which is closely related to microcrack formation. The variety in the structure of dentin obtained from the tooth bank and factors such as age continue to exist in the scientific world as a limitation of in vitro studies. Therefore, in this study, brushing motion and pecking motion were analyzed in the same sample by virtue of the fact that the tendency of microcrack formation would be the same.

Today, root canal instrumentation can be performed with many engine-driven file systems with various properties in kinematics, dimensions, alloy, etc [4, 19–21]. Manufacturers have introduced various single or multi-file systems with rotational or reciprocal kinematics. They show superiority in various canal morphologies due to their unique advantages [10, 18]. As file systems have taken different forms in various generations, questions regarding the properties and efficiency of these file systems have remained on the agenda. The formation of microcracks is notable as one of these properties. The aim of the study is to evaluate the microcrack formation of rotary and reciprocal multi- and single-file systems when used with brushing motion on canals. The null hypothesis is that brushing motion does not contribute to additional microcrack formation.

## Materials and methods

### Sample selection

The method and protocol of the study were approved by the local Research Ethics Committee (protocol #70904504/917). The study was conducted according to the guidelines outlined in the Declaration of Helsinki, version 2008. The sample size of the study was determined according to previous data [22] with a power of 95%, a confidence level of 0.05, and an effect size of 0.75 using the software G*Power 3.1 (Heinrich–Heine–Universität, Düsseldorf, Germany). The required minimum sample size was 11 for each group, and 1 sample was added to each group for laboratory losses. Thus, the sample size of the study was 12.

Mandibular molars were collected from a private dental clinic, where the patients opted for tooth extraction for various reasons unrelated to the study including chronic periodontitis, carious teeth with apical symptomatic or asymptomatic apical periodontitis, and abscess. The specimens were obtained from patients aged between 28 and 39 years. Oral and written informed consent was obtained.

Inclusion criteria included mandibular molars with two distinct roots, and moderately curved canals (10°-20°) according to Schneider [27]. To determine the curvature of the canal, periapical radiography was taken in a buccolingual direction. Exclusion criteria were root caries, root resorption, teeth with calcified canals, teeth with root canal treatment, filling, crown or endocrown prosthesis, C-canal morphology, radix entomolaris, and third molars Root lengths were standardized at 13 ± 2 mm from the orifice to the apical. Selected teeth were examined using a stereomicroscope under x12 magnification in terms of the presence of cracks or any resorption.

Mandibular molar teeth with a distal root with a round canal (similar values for buccolingual and mesiodistal diameters) and a mesial root with a Vertucci type IV canal morphology were selected for the study. To detect Vertucci canal configuration, and further crack or fracture investigation, a cone-beam computed tomography (CBCT) image of each sample was taken using NewTom VGi Evo (QR, Verona, Italy) with exposure parameters of 0.3 mm voxel size, 110 kV, 32 mA, 7.3 s of exposure time, and 10 × 10 mm field of view. CBCT scanning of mandibular molars was performed with a silicon mold to ensure immobilization and a stable vertical position. Axial, sagittal, and coronal sections were analyzed. After analyzing radiographic images, according to inclusion criteria, 36 specimens were picked out. Since the microcrack tendency of the specimen changes with dehydration until the laboratory process, extracted teeth were kept at 3% sodium hypochlorite for 15 min followed by a 0.1% thymol solution at 4 °C for up to 3 weeks [28].

### Obtaining of micro-CT images

Since humidity adversely affects the image, before scanning the samples were dried gently and kept in a dry environment for 2 h, a period that would not cause new microcracks [23, 29, 30]. Preoperative micro-CT scans of selected specimens were performed by a micro-CT (SkyScan 1173; Bruker microCT, Kontich, Belgium) using a custom-prepared mounting device. The exposure parameters of the micro-CT were isotropic resolution of 11.25 μm with 360° rotation around the vertical axis of the tooth, rotation step of 0.5°, 7000 ms of exposure time, frame averaging of 5, and filtered with the 1 mm thick aluminum. During scanning, the roots of samples were in an upward position. Images were investigated by NRecon v.1.6.10 software (Bruker micro-CT, Kontich, Belgium) with ring artifact correction of 10 and beam hardening correction of 40%. Scanning and analyzing parameters were stabilized for all samples. In preoperative micro-CT images, Vertucci type IV canal morphology in the mesial root, and one round canal in the distal root was confirmed. To correctly analyze and evaluate the presence of beam hardening or ring artifacts an oral and maxillofacial radiologist analyzed all micro-CT images (X.X., a 6-year oral and maxillofacial radiologist).

### Allocation of the samples

After scanning micro-CT, 36 specimens were randomly allocated to three study groups (*n* = 12) according to the instrument system using the algorithm (http://www.random.org) and coded with a binary letter system blinded to the observers that going to perform root canal preparation and micro-CT analysis. Three groups were.

### Group MFR-P

Multi-file rotary system (Protaper Next) applied to the mesial canals with only pecking motion (*n* = 12).

#### Group MFR-B

Multi-file rotary system (Protaper Next) applied to the distal canal with pecking motion and brushing motion (*n* = 12).

#### Group SFR-P

Single-file rotary system (TruNatomy) applied to the mesial canals with only pecking motion (*n* = 12).

#### Group SFR-B

Single-file rotary system (TruNatomy) applied to the distal canal with pecking motion and brushing motion (*n* = 12).

#### Group SFRc-P

Single file reciprocal system (WaveOne Gold) applied to the mesial canals with only pecking motion (*n* = 12).

#### Group SFRc-B

Single file reciprocal system (WaveOne Gold) applied to the distal canal with pecking motion and brushing motion (*n* = 12).

Samples divided into groups were prepared using the file systems specified according to the groups; the mesiobuccal and mesiolingual canals were shaped with pecking motion, and the distal canals were shaped with pecking motion in addition to brushing motion.

##### Root canal preparation

Traditional endodontic cavities were performed. In mandibular molars, apical patency was obtained using the #10 K-file. The initial apical file was confirmed to be no larger than ISO #10, and two independent canals and apical foramina in the mesial root were checked, subsequently, the file was inserted into the canal until the tip was visible and the length was measured. The working length was calculated by subtracting 0.5 mm of the length measured. To mimic bone, periodontal ligament, and apical gas entrapment, the apex of the root was sealed with glue, and the root was embedded in polyvinyl siloxane and acrylic resin to cementoenamel junction and kept in humid conditions until the instrumentation procedures. To standardize the periodontal ligament, a thin plaster band was wrapped in a single layer around the root of each sample and after acrylic polymerization, the plaster was removed, the roots of the samples were coated with silicone and placed in the acrylic socket. To simulate the oral conditions, mandibular molars were kept in a heat-controlled water bath at 37 °C all the process of instrumentation. Before engine-driven instrumentation, a #15 K-file was used to prevent the breakage of the instrument and the taper-lock effect. During all engine-driven and manual instrumentation, the pulp chamber was filled with 5% NaOCl.

##### Group MFR-P and group MFR-B

In these groups, the Protaper Next file system was used. Proglider (apical diameter/taper: 0.16/0.02), as a pathfile, was used according to the manufacturer’s instructions to all canals of mandibular molars. In this multi-file rotary system; X1 (apical diameter/taper: 0.17/0.04 taper) and X2 (apical diameter/taper: 0.25/0.06 taper) were used, in sequence. Instrumentation was performed under a continuous clockwise rotation at 300 rpm and 4 N/cm^2^ torque [31] using an endomotor (X-Smart Plus, Dentsply, Maillefer, Ballaigues, Switzerland) with a 16:1 contra angle.

##### Group SFR-P and group SRF-B

In these groups, the TruNatomy file system was used. TruNatomy Glider (apical diameter/taper: 0.17/0.02), as a pathfile, subsequently, the TruNatomy Prime file (apical diameter/taper: 0.26/0.04, variable taper) was used at 500 rpm and 1.5 N/cm^2^ torque using the same endomotor.

##### Group SFRc-P and group SRFc-B

In these groups, the WaveOne Gold file system was used. WaveOne Gold Glider (apical diameter/taper: 0.15/0.02), as a path file, was used followed by WaveOne Gold Primary file (apical diameter/taper: 0.25/0.07 variable taper), which was placed into the canal, activated, and canals were instrumented at “WaveOne Gold” mode of the same endomotor.

**Fig. 1:**
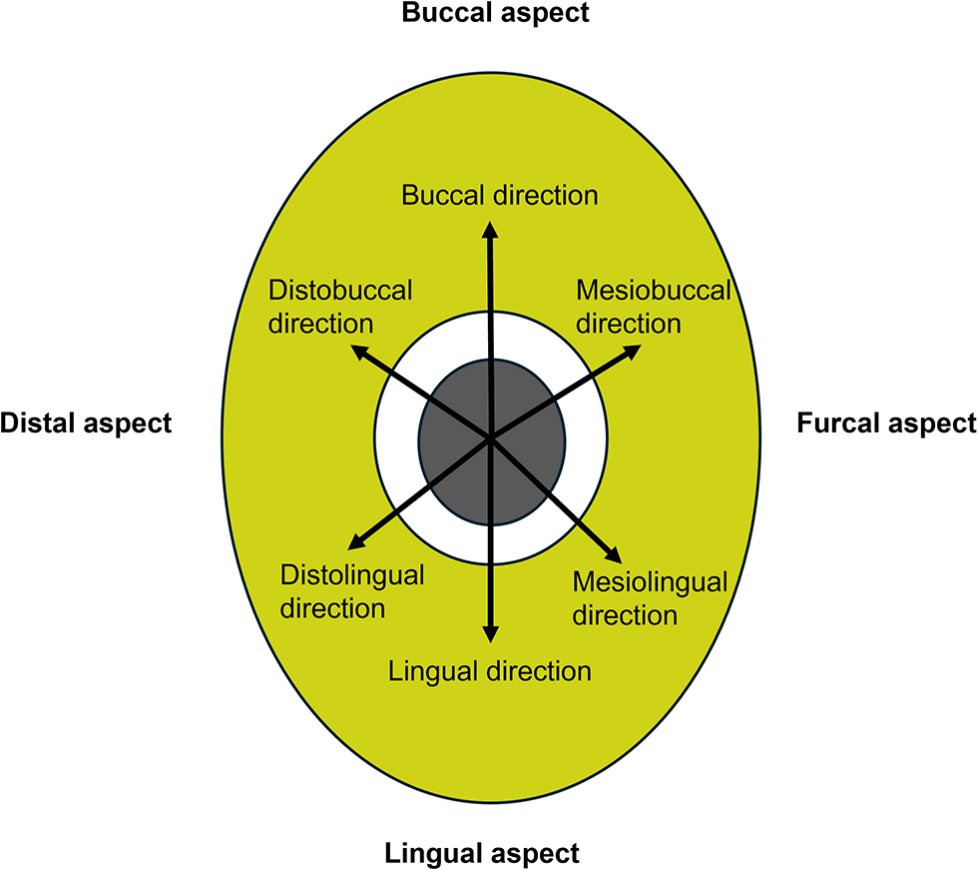
Representative schema about applying the method of brushing motion

For all groups, in the mesial canals, up and down pecking movements with an amplitude of 3 mm were performed until reaching the working length. To standardize the amplitude of motions, instrumentation was performed in 3 mm sections using a plastic stopper of a #6 K-file. In the distal canal, the pecking motion was applied similarly to that used in mesial canals, in addition to this, whereafter reaching the working length a systematic brushing motion with 6 strokes was performed in buccal, mesiobuccal, mesiolingual, lingual, distolingual, and distobuccal directions, in the similarly clockwise direction by pulling the file 1–2 mm in all samples (Fig. 1). The instrumentation duration of each sample was stabilized to 3.5 min excluding irrigation time using a digital chronometer. Recapitulation was performed using a #15 K-file between pathfiles and shaping files in every group, and between shaping files in MFR-P and MFR-B groups. A new file was used for each canal. During instrumentation, between rotary or hand files, irrigation with 5 mL 5.25% NaOCl was applied for 1 min by the NaviTip irrigation needle (Ultradent Products, Inc., South Jordan, UT, USA) that was inserted up to 2 mm from the working length and at a rate of 1 mL/min [32]. As a final irrigation protocol, canals were irrigated with 5 mL 5.25% NaOCl and 5 mL 17% EDTA by stabilizing the total volume of irrigation solution for each specimen. Following the final irrigation, canals were dried with paper points in proper dimensions, and to verify the instrumentation, the proper gutta-perchas according to the file systems, were inserted in the root canal until reaching the working length. After checking the shaping of the canals, each canal was dried using a paper point one more time.

Chemomechanical instrumentation was performed by one operator (D.Y., 9 years of experience using NiTi files) with calibrated motions in pressure and speed, who was blind to preinstrumentation scans and the binary coding of the samples.

As in the pre-instrumentation scans, before scanning the samples were dried gently and kept in a dry environment for 2 h [23, 29, 30]. Post-instrumentation micro-CT scans were obtained with similar parameters to pre-instrumentation scans.

### Analysis of micro-CT scans

Microcracks were classified as (1) incomplete cracks that extend from the root canal wall through the dentin without reaching the outer surface, (2) complete cracks that extend from the root canal wall to the outer surface, and (3) craze lines that present in dentin itself without reaching the outer surface or root canal. Microcrack evaluation in pre- and post-instrumentation micro-CT scans was performed with two operators independently and twice. Operators (D.Y. and S.O.) were blind to the binary code of groups. A 4-week intermission was taken before the second evaluation by the same operator. During the post-instrumentation microcrack evaluation, the detected microcracks were confirmed in the same pre-instrumentation scans [10]. In case of disagreement among researchers regarding the microcrack report, cross-sectional analyses were continued until agreement was reached. Before the micro-CT analysis, to obtain optimal visualization, contrast or brightness values were adjusted by the image tools of the software, and all examinations were performed in a dark room.

### Statistical analysis

Statistical analysis was carried out by SPSS version 22.0 (IBM Corp.,Armonk, NY, USA). The percentages of microcracks in pre- and post-instrumentation scans were calculated. Shapiro–Wilk test and Levene’s tests were used for the normality and homogeneity of the data, respectively. The difference in microcracks between pre- and post-instrumentation scans was analyzed using the Wilcoxon signed-rank test. Mann Whitney U test was used to compare the pecking and brushing motions. The effect of kinematics was analyzed using the Kruskal-Wallis test. Interclass correlation coefficient (ICC) was used for intraobserver and interobserver reliability. The level of significance with a 95% confidence interval was set at *p* < 0.05. Values with a difference of *P* < 0.001 were considered statistically significant for ICC analysis.

## Results

**Fig. 2:**
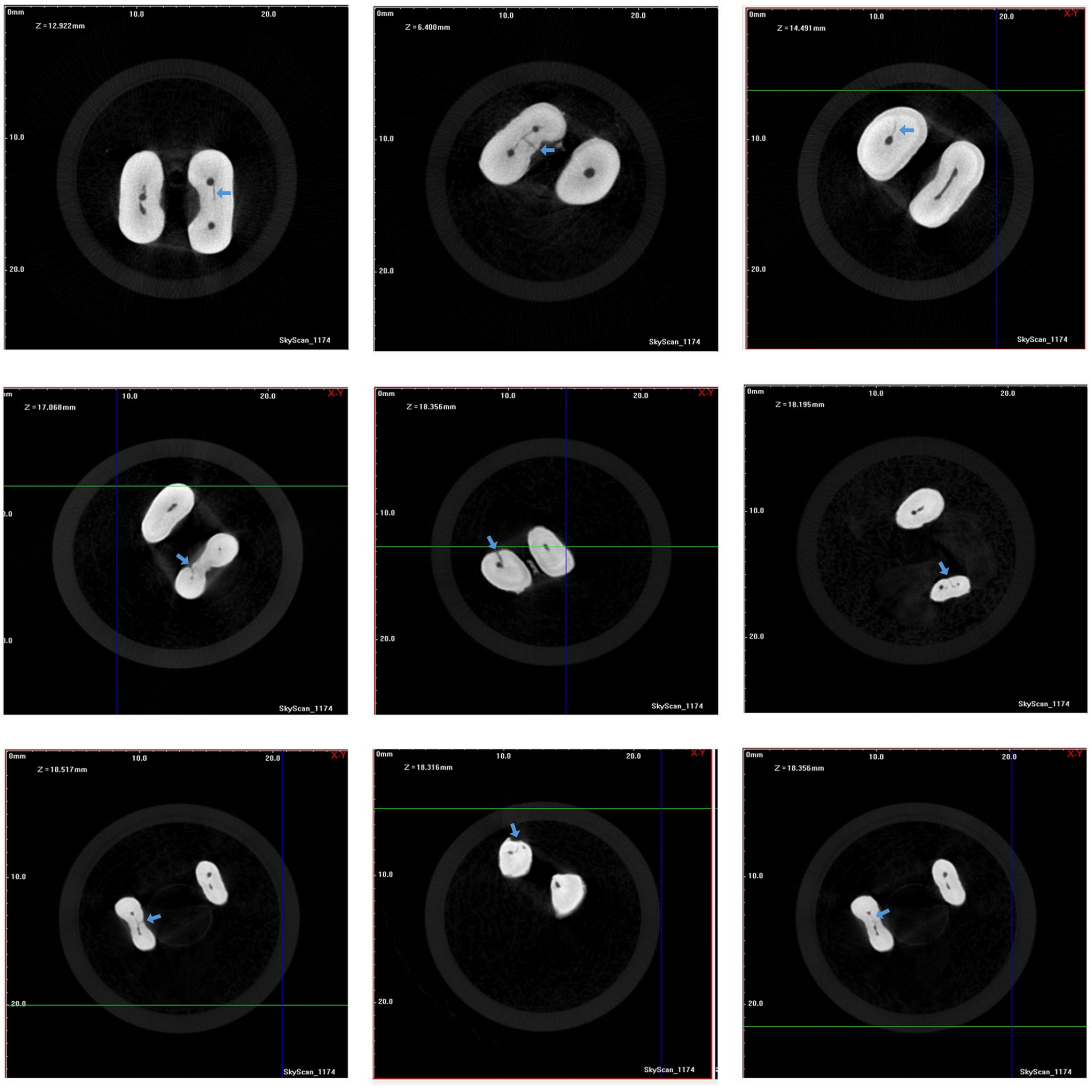
Microcracks on samples on different levels

A total of 46 256 cross sections were generated in pre- and post-instrumentation micro-CT. During the instrumentation, no instrument fractures, root fractures, or loss of working length occurred requiring specimens to be excluded from the study. In microcrack analysis, no discrepancy was observed between the two observers in terms of the presence of microcracks. Tables 1 and 2 show the microcrack percentages in pre and post-instrumentation scans belonging to file systems. According to Wilcoxon analysis, there was no statistical difference between pre-and post-instrumentation micro-CT images in terms of the presence of microcracks in any file system groups (*p* > 0.05). No statistical difference between Group MFR-P, Group SFR-P, and Group SFRc-P, and similarly Group MFR-B, Group SFR-B, and Group SFRc-B, (*p* > 0.05). The kinematics and the number of files did not affect the microcrack formation. According to the Whitney U test, no difference was detected between Group MFR-P and Group MFR-B, Group SFR-P, and Group SFR-B, and Group SFRc-P and Group SFRc-B (*p* > 0.05). The brushing motion that was followed by the pecking motion did not cause an extra microcrack. Figure 2 shows the microcrack on the samples.

**Table 1:** In-and-out pecking motion on microcracks (%) in mesiobuccal and distobuccal canals of mesial root (PTN: protaper next, WOG: WaveOne gold, TRN: TruNatomy)

		Pre-instrumentation (%)	Post-instrumentation (%)	*P* Value
**Group MFR-P**	Coronal	8.14	8.18	0.15
	Middle	9.74	9.79	0.64
	Apical	12.5	12.52	0.19
**Group SFRc-P**	Coronal	8.65	8.68	0.45
	Middle	11.57	11.61	0.09
	Apical	21.64	22.13	0.30
**Group SFR-P**	Coronal	15.69	15.90	0.16
	Middle	12.35	13.05	0.18
	Apical	10.72	10.72	0.34

No statistical difference between pre and post-images according to Wilcoxon analysis (*p* < 0.05)

(MFR-B: multi-file rotary-pecking. SFRc-B: single-file reciprocal-pecking. SFR-B: single file rotary pecking)

**Table 2:** In-and-out pecking motion following brushing motion on microcracks (%) in mesiobuccal and distobuccal canals of mesial root

		Pre-instrumentation (%)	Post-instrumentation (%)	*P* Value
**Group MFR-B**	Coronal	14.23	14.54	0.056
	Middle	9.16	12.27	0.14
	Apical	10.47	10.59	0.06
**Group SFRc-B**	Coronal	7.98	8.06	0.38
	Middle	9.64	10.23	0.07
	Apical	13.24	14.06	0.14
**Group SFR-B**	Coronal	9.24	9.29	0.39
	Middle	11.63	11.63	0.06
	Apical	15.64	15.68	0.31

No statistical difference between pre and post-images according to Wilcoxon analysis (*p* < 0.05)

(MFR-B: multi-file rotary-brushing. SFRc-B: single-file reciprocal-brushing. SFR-B: single file rotary brushing)

The ICCs were excellent, and the values were between 0.941 and 0.987 for the intraclass analysis. For interclass analysis, the values were between 0.934 and 0.985, excellent.

## Discussion

This study examined for the first time the effect of brushing motion on microcrack formation and found that brushing motion followed by pecking motion did not cause additional microcrack in the round root canal using multi-file rotary (ProTaper Next), single-file rotary (TruNatomy), and single-file reciprocal (WaveOne Gold) file systems. The results support the null hypothesis that brushing motion does not contribute to additional microcrack formation.

In the last published critical analysis on microcracks, 60% of the studies using the micro-CT method reported that no new microcracks were formed with engine-driven instrumentation [7]. Almost all of these studies applied pecking motion during instrumentation [8–10, 12, 13, 18, 22, 23, 33, 34]. A few studies have reported a “slightly brushing motion’ and ‘brushing coronal two-thirds’ [11, 21]. As a supplementary movement, brushing motion is applied with lateral pressure during mechanical instrumentation to enhance microbial control and provide a higher biofilm removal, in addition to all irrigation and irrigation activation protocols [4]. The microcracks that this lateral pressure can create have not been systematically studied before. For the examination of the effect of brushing motion, detailed information about the direction and amount of brushing motion is required. In this study, a total of 6 brush strokes were applied in the buccal, lingual, mesiobuccal, mesiolingual, distobuccal, and distolingual directions, and to standardize the brushing movement. No difference was detected pre- and post-instrumentation microcracks with additional brushing motion. Since in clinical practice, it does not seem possible to apply brushing motion alone without following the pecking motion in the apical direction, brushing motion was applied as an additional brushing method to the pecking motion in this study.

Studies have analyzed before and after microcrack formation via micro-CT images. While most studies reported no new microcracks after instrumentation [10–13, 23, 33], some of them stated the formation of new microcracks [8, 9, 22, 34]. In our study, the effects of rotary and reciprocation systems on the formation of dentinal microcracks were examined. There are discrepancies in the literature about the effect of kinematics. Pop et al. [19] found no difference between kinematics. Another study showed that rotary instruments produce more microcracks than reciprocating motion [18]. However, in these studies, while muti-file files were used in the rotary system, a single file was used in reciprocation. It can be concluded that this difference is related to the number of files as well as kinematics. In our study, a single file was used in reciprocation and rotary kinematics, and the effect of the kinematics could be interpreted more clearly.

The relationship between microcrack formation and engine-driven instrumentation in endodontic literature is poor [7]. However, it is important to interpret the details in the analysis of microcrack formation. Most of the studies that analyzed microcracks as present or absent did not measure their dimensions and did not provide data on whether there was an increase in the length of microcracks after instrumentation. Instrumentation can cause the formation of new microcracks, as well as an increase in the size of existing cracks. Unfortunately, the designs of existing studies analyzed the presence of microcracks qualitatively and did not report changes in the dimensions of microcracks. At the point where instrumentation does not cause the formation of new microcracks, it may cause an increase in the dimensions of existing microcracks. Therefore, it may not be accurate to conclude that instrumentation and microcrack phenomena are unrelated, based on currently available scientific evidence. In the literature, only one study measured the length of microcracks at 5 μm voxel size [35], and this study reported that a single-file rotary system increased the length of microcracks. Our study also did not measure the length of existing microcracks. Further studies should focus on the effect of brushing motion with low voxel size on the dimensions of existing microcracks. On the other hand, it has been reported that some microcracks turn into complete cracks using some rotary or reciprocating file systems that do not create new microcracks [36]. In that study comparing the XP-endo Shaper and Reciproc Blue systems, it was shown that the Reciproc Blue had a greater effect on the expansion of microcracks than the XP-endo shaper [36]. In two systems with different kinematics and the same number of files, according to our results, it was seen that kinematics did not constitute a difference in microcrack formation.

Over time, the scientific method for microcrack evaluation evolved from the section method to micro-CT analysis using cadavers or extracted teeth by acceptance of the scientific community [7, 12]. In section analysis, the sectioning process may cause the formation of new microcracks, in addition to this, the analysis allows only a few sections to be examined. On the contrary, while thousands of sections are examined with micro-CT since it is not a destructive method, no procedure is applied that may result in the formation of new microcracks in the teeth after instrumentation. The non-destructive nature of micro-CT provides specimens of the study to be their own control group with longitudinal observation, which is a more reliable scenario compared to the independent control group as in the section analysis. Since the limitations of the section method have been clearly demonstrated [7], the results of this study were not compared with those of previous studies using section analysis.

In micro-CT analysis, one of the most important factors affecting the results is irradiation parameters including voxel size. Studies have examined the presence of microcracks at a resolution ranging from 5 to 33 μm [8–10, 36]. The detection of microcracks depends on the voxel size. Pinto et al. [24] demonstrated that a 5 μm voxel size revealed cracks that were undetectable at 10–20 μm. Our study used an 11.25 μm voxel size, which may have influenced detection sensitivity, underestimating their presence.

In addition to voxel size, another effective factor in detecting microcracks is the presence of moisture. Previously it was reported that moisture in the samples could block microcracks in the image [29, 30]. However, a balance must be obtained between the detectability of existing microcracks and the formation of new ones due to overdehydration. That study reported that drying for 4 h could result in new microcracks [30]. Furthermore, dry periods of up to 24 h did not induce microcrack formation [29]. Martins et al. [23] reported no new microcracks in 2 h of drying. Therefore, in our study, we allowed the samples to 2 h of drying at room temperature.

Due to the aging process, changes occur in the biochemical structure of collagen including the formation of cross-links and advanced glycation end-products, accordingly, the stiffness, flexibility, and tenderness to the microcrack formation of the collagen evolves. Since dentin contains abundant collagen in its structure, the age of the extracted tooth samples collected is crucial for the standardization of the study. Literature has shown that older ages (40–70 years) are more prone to microcrack formation than younger people (20–39 years) [37]. Therefore, knowing the age group of the samples in studies analyzing microcrack formation may have an impact on the results. However, some of the studies do not provide any data on age [8, 9, 11, 13, 22–24, 36]. On the other hand, studies conducted on cadavers were carried out in two age ranges; 19–30 [12], and 64–99 [29]. Research conducted on extracted teeth reported an age range limited to 15–20 years [21, 30], while one of them reported a wide range of ages (20–70 years) [35]. Our study limited the age of samples to between 28 and 39 to enhance the standardization. For our study, it is worth noting that the effect of pecking motion and brushing motion was analyzed in different roots of the same sample, thus, the parameters related to dentin structure were already stabilized. For a better-standardized analysis of different file systems, the age period was limited to 28–39 years.

In the study, in order to mimic the clinical conditions properly, both mesiobuccal and mesiolingual canals in the mesial root where pecking motion was applied were instrumented, as previously reported [22]. The effect of brushing motion on the intercanal dentin during the instrumentation of two mesial canals close to each other should be investigated with further studies.

The findings of the study suggest that brushing motion can be safely incorporated into endodontic instrumentation without increasing the risk of dentinal crack enhancement. Since it does not increase the presence of microcracks, brushing motion is recommended to be added to the instrumentation protocol in the presence of advanced infection in oval canals to improve mechanical microbial control. On the other hand, the presence of thin dentin such as the danger zone should be taken into consideration.

The limitation of this study is that it was able to examine microcracks within a voxel size of 11.25 μm. Further studies should be established with lower voxel size and a detailed microcrack analysis including the length of the crack. This study evaluated file systems with different tapers and diameters. This difference should be considered as a limitation in the interpretation of the results. Besides, this study used teeth extracted for various reasons. Although pre-instrumentation and post-instrumentation microcracks were analyzed, it is recommended that extraction techniques be standardized in future studies. On the other hand, we would like to remind readers that this study is in vitro and cannot fully mimic in vivo conditions. The strength of the study is that it systematically examines the effect of brushing motion on microcrack formation under standardized strokes. On the other hand, since the effects of brushing motion and pecking motion on microcrack formation were performed on the same sample, differences depending on the dentin structure (tooth age, collagen ratio, chemical content, etc.) were also standardized. It may be attributed as a strength of the study.

## Conclusion

Considering the limitations of the study, it can be concluded that the additional brushing motion did not cause microcrack on round canals after using multi-file rotary (ProTaper Next), single-file rotary (TruNatomy), and single-file reciprocation (WaveOne Gold) systems. It was observed that none of the file systems analyzed in this study increased the microcracks when examined at a voxel size of 11.25 μm via micro-CT.

## Electronic supplementary material

Below is the link to the electronic supplementary material.

Supplementary Material 1

## Data Availability

No datasets were generated or analysed during the current study.
