# Supplementary figures and images for: Brushing motion caused no microcracks: a micro-computed tomography study
Source: Clin Oral Investig. 2025 Mar 10;29(3):173. doi: 10.1007/s00784-025-06253-0 (PMC11893623; doi:10.1007/s00784-025-06253-0)

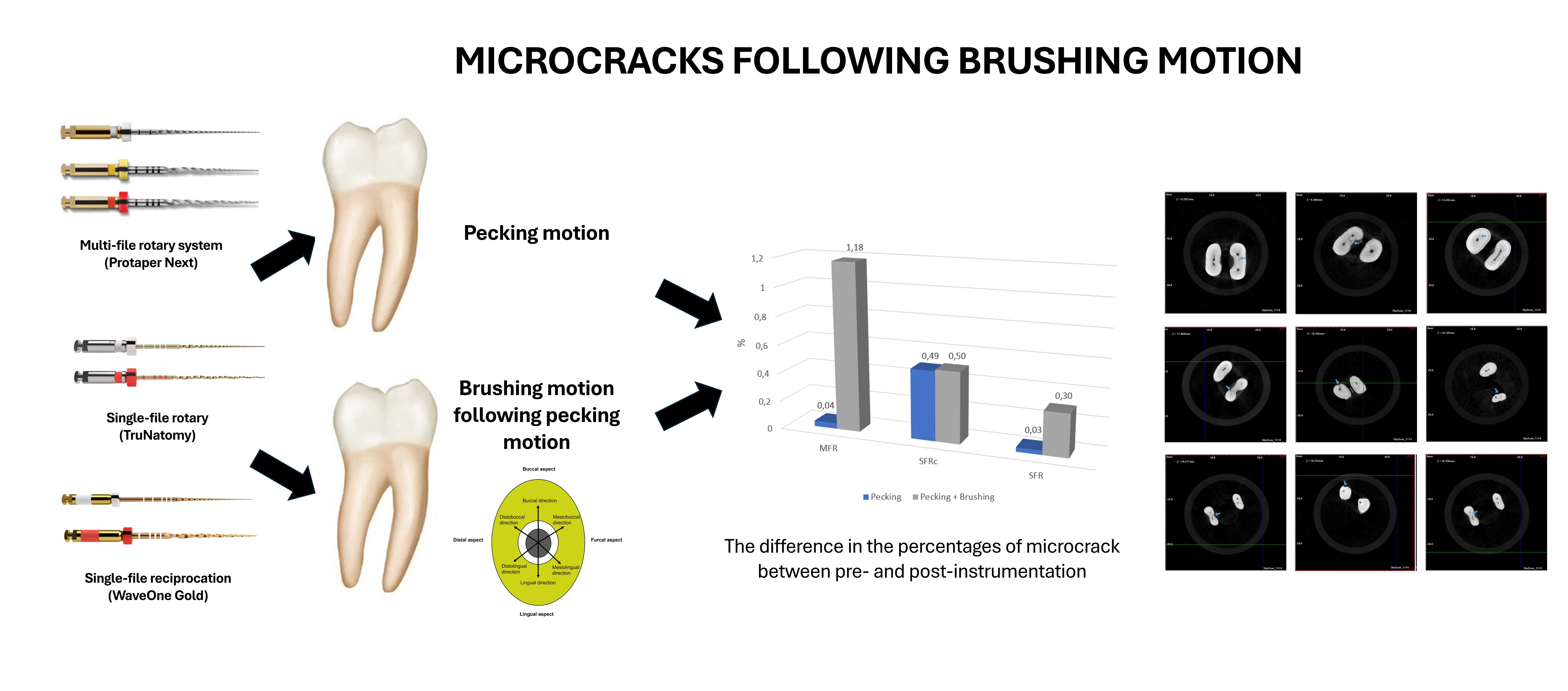

Supplement: Supplementary file 1 — Supplementary Material 1 [file 784_2025_6253_MOESM1_ESM.png]
